# Endoscopic features and clinical course of patients with asymptomatic cecal ulcers

**DOI:** 10.1186/s12876-022-02383-x

**Published:** 2022-06-24

**Authors:** Ying-Cheng Lin, Szu-Chia Liao, Chung-Hsin Chang, Chia-Chang Chen, Wan-Tzu Lin, Fang-We Chiu, Chung-Wang Ko

**Affiliations:** 1 Division of Gastroenterology and Hepatology, Department of Internal Medicine, Taichung Veterans General Hospital, 1650, Section 4, Taiwan Boulevard, Xitun Dist., Taichung City, 40705 Taiwan; 2 School of Medicine, College of Medicine, National Chung Hsing University, Taichung, Taiwan; 3 Division of Gastroenterology and Hepatology, Department of Internal Medicine, Puli Branch of Taichung Veterans General Hospital, No. 1, Rongguang Rd., Puli Township, 54552 Nantou Taiwan; 4 School of Medicine, College of Medicine, National Yang Ming Chiao Tung University, Taipei, Taiwan

**Keywords:** Cecal ulcer, Endoscopy, Asymptomatic, Intestinal tuberculosis, Crohn’s disease, Ulcerative colitis, Inflammatory bowel disease

## Abstract

**Background:**

Cecal ulcers are sometimes encountered in asymptomatic individuals. Their clinical outcomes and management recommendations remain uncertain.

**Methods:**

Asymptomatic patients who underwent a colonoscopic exam for colon cancer screening were retrospectively reviewed from July 2009 to November 2016. Patients with cecal ulcers were included. Patients who had colorectal symptoms, such as abdominal pain, had nonsteroidal anti-inflammatory drugs or were lost to follow-up were excluded.

**Results:**

A total of 34,036 patients underwent colon cancer screening. Cecal ulcers were found in 35 patients. After exclusion, 24 patients (mean duration, 52 months) received follow-up colonoscopy. In 20 patients, (83.3%), cecal ulcer resolved without intervention, but 4 patients (16.7%) developed clinical significant diseases, including intestinal tuberculosis (n = 2), Crohn’s disease (n = 1), and ulcerative colitis (n = 1). Patients who developed clinically significant diseases had a higher percentage of ulcers larger than 1 cm (75% vs. 15%, *p* = 0.035), terminal ileum involvement (100% vs. 15.4%, *p* = 0.006) and ulcers with irregular fold (75% vs. 5%, *p* = 0.008).

**Conclusions:**

In patients with asymptomatic cecal ulcers, the endoscopic features included larger ulcer size, terminal ileum involvement and ulcers with irregular fold may predict development of clinically significant diseases. If the above-mentioned features are present, even asymptomatic patients should be closely monitored.

## Background

Cecal ulcers can be encountered in a variety of diseases including inflammatory bowel disease, infectious colitis and malignancy. Previous reports have investigated patients with gastrointestinal symptoms, such as acute lower gastrointestinal bleeding and right lower abdominal pain. The diagnosis may be inconclusive even after biopsy [1]. For urgent cases, such as patients with profound bleeding, surgical intervention may be necessary for a definite treatment and diagnosis [2, 3]. Since the preoperative diagnosis is sometimes difficult, close follow-up has been suggested in these cases, which were managed conservatively [1, 4].

Colonoscopic screening programs are performed for healthy adults worldwide. In the United States, for example, 15 million colonoscopic exams were performed (more than 40% for the purpose of cancer screening) and this number is rising each year [5]. Cecal ulcers are sometimes encountered in asymptomatic patients. Whether those patients represent the earliest course of a clinically significant disease has not been evaluated. The clinical and endoscopic characteristics that may predict disease development, such as inflammatory bowel disease, are largely unknown. Herein, we aimed to investigate the long-term outcomes of cases with asymptomatic cecal ulcers and to analyze the endoscopic features that predict future disease.

## Methods

### Study design

Patients who underwent colonoscopic screening at the health promotion center of Taichung Veterans General Hospital from June 2009 to December of 2016 were retrospectively analyzed. Clinical outcome was followed until December, 2019. The patients who visited the health promotion center were interviewed by a general practitioner and their medical history, including drug exposure and recent health condition, were recorded before the colonoscopic exam. Basic laboratory exams including white blood cell, hemoglobin level, CRP, renal function, and HbA1c were also obtained on the same day and before colonoscopic exam.

### Patient population

Patients with a diagnosis of ulcer in their colonoscopic report were identified. Cecal ulcer was defined as mucosal breaks over the ileocecal valve or cecum. Patients with erosions without true mucosal breaks or only hyperemic changes were excluded. In addition, any patients (1) with a history of nonsteroidal anti-inflammatory drug (NSAID) usage 1 month before; (2) with symptoms of colorectal disease, such as recent abdominal pain; (3) who remained asymptomatic but refused to have a repeat colonoscopic exam in the out-patient department were also excluded. Medical charts, personal history, laboratory, endoscopic findings, and histologic reports were reviewed. All of the patients received polyethylene glycol solution for bowel preparation. All initial colonoscopies were performed with gastroenterologists using standard endoscopes. Endoscopic images were reviewed by two authors (Ko and Liaw), who were blinded to the patients’ clinical results.

### Endoscopic definition

Ulcers with irregular fold were defined as ulcers that resulted in loss of the normal haustral folds or fold convergence [6, 7]. Terminal ileum involvement was defined by the presence of ulcers in the terminal ileum in those with asymptomatic cecal ulcers. Size of ulcers were estimated based on the largest ulcer. Due to the retrospective nature, the precise numbers of ulcers were difficult to determine and were estimated by the number of available lesions in colonoscopic photographs. If discrepancies existed between the two endoscopists in their evaluation of the presence of ulcer with irregular fold or the size and numbers of cecal ulcer, a third endoscopist (Chiu) was consulted.

### Statistical analyses

Categorical variables are presented with numbers (percentages) and were compared by Chi-Square test. Continuous variables are presented with median (interquartile range) and were compared using Fisher’s exact test. *p* Values < 0.05 were considered statistically significant. Multivariate logistic regression analysis was used to evaluate the odds ratio (OR) and 95% confidence interval (CI) of risk factors for cecal ulcer. Statistical tests were performed using the Statistical Package for the Social Sciences (IBM SPSS version 22.0; International Business Machines Corp, New York, USA). This study was approved by the Institutional Review Board of Taichung Veterans General Hospital (CE17350A). Because all data were de-identified prior to analysis, the requirement for informed consent from the participants was waived.

## Results

**Fig. 1:**
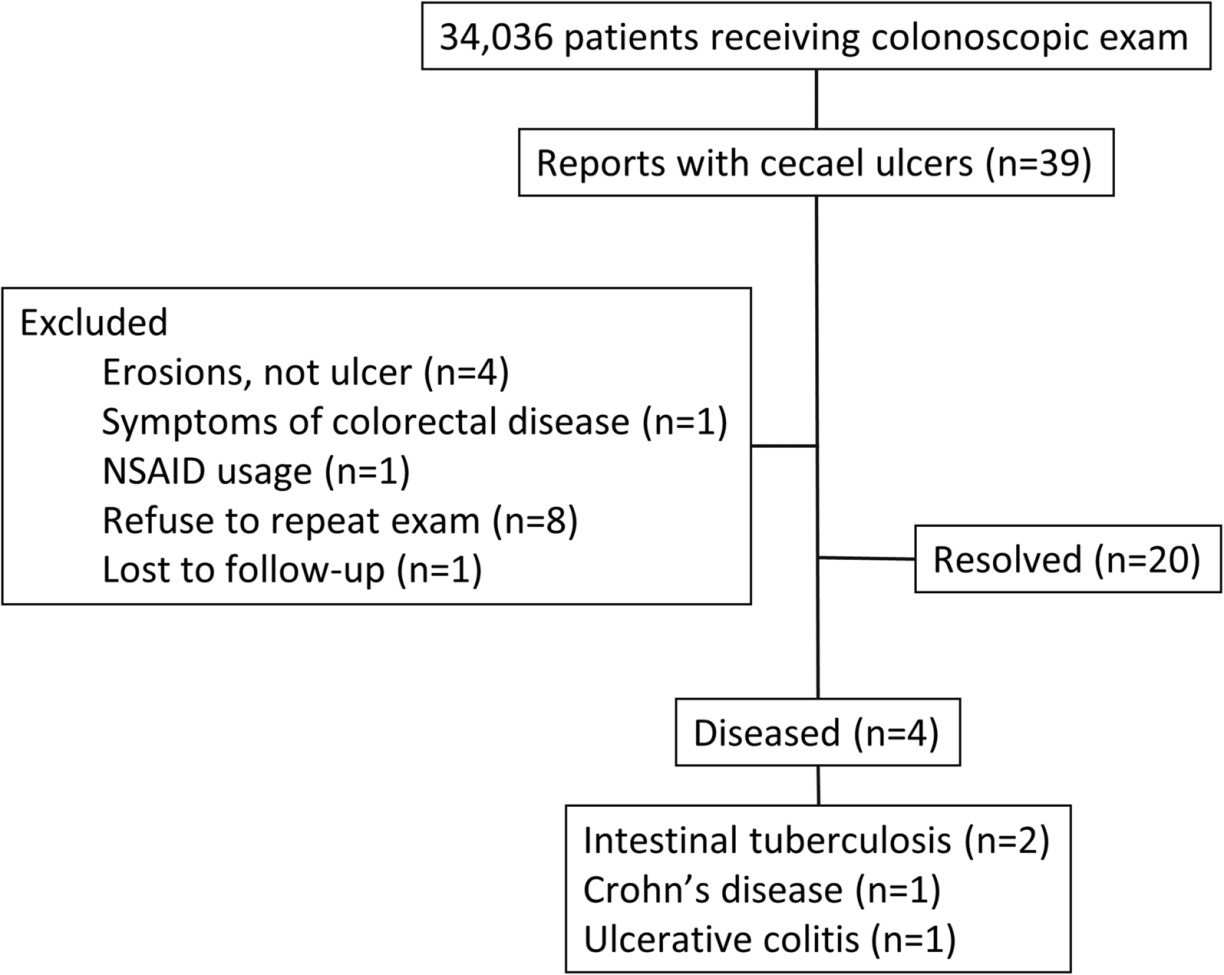
Flowchart of participant recruitments and outcomes. *NSAID* non-steroidal anti-inflammatory drug

The flowchart of patient recruitment is shown in Fig. 1. A total of 34,036 patients underwent colonoscopic screening in the health promotion center during June 2009 to December 2016. Among those screenings, cecal ulcers were identified in 39 patients. After excluding those without definite ulcer (n = 4), symptoms of colorectal disease (n = 1), recent NSAIDs usage (n = 1), refusal to undergo repeat colonoscopy (n = 8), and loss to follow-up (n = 1), 24 patients had at least one follow-up colonoscopy.

### Patient characteristics

Twenty-four patients, consisting of 14 males and 10 females, had a median age of 52.7 years, ranging from 25.8 to 69.7. The basic demographic characteristics of patients with resolved disease or progression were not significantly different in age, gender, smoking status and other laboratory findings, including white blood cell, hemoglobin level, CRP, renal function and HbA1c (Table 1). Of note, all 24 patients with asymptomatic cecal ulcers had normal hemoglobin level. Only one patient presented with significantly elevated CRP (4.79 mg/dL, with normal range < 0.3 mg/dL). There were no differences in age, gender, or laboratory data between the 24 patients who had at least one follow-up colonoscopy and those 8 patients who refused to undergo a repeat colonoscopy (data not shown).

**Table 1:** Demographic characteristics of patients with asymptomatic cecal ulcers

	Resolved (n = 20)	Progression to disease (n = 4)	*p* value
Age	50.40 (25.8–69.7)	53.35 (52.7–59)	0.304
Gender			0.272
Female	7 (35.00%)	3 (75.00%)	
Male	13 (65.00%)	1 (25.00%)	
Lab data			
WBC (/µL)	6020.00 (3000–12,400)	7685.00 (3960–12,500)	0.785
Hemoglobin (g/dL)	14.9 (12.8–16.5)	14.9 (13.6–15.8)	0.631
CRP (mg/dL)	0.14 (0.01–0.79)	1.22 (0.01–4.79)	0.237
Creatinine (mg/dL)	0.91 (0.46–1.29)	0.78 (0.64–1.09)	0.491
HbA1c (%)	5.80 (5.4–6.2)	5.55 (5.4–8.8)	0.608
Guaiac-based FOBT	Negative (19), Trace (1)	Negative (4)	NA
Tobacco smoking	5 (25.00%)	0 (0.00%)	0.544

Continuous data are expressed as median (Range). Categorical data are expressed as number and percentage

*WBC* white blood cell, *CRP* C-reactive protein, *FOBT* fecal occult blood test, *NA* not available

### Endoscopic features

**Fig. 2:**
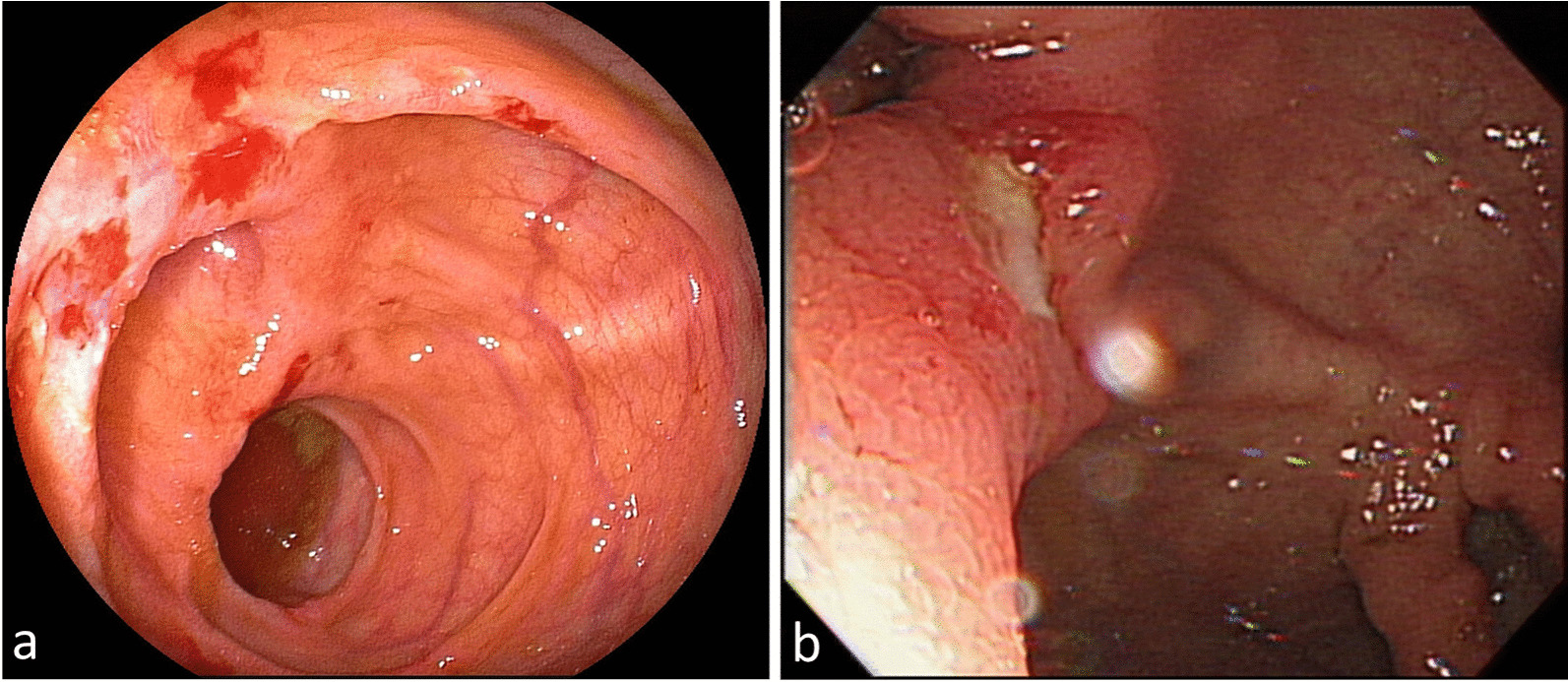
Cecal ulcers with irregular fold. **a** The patient was later diagnosed as Crohn’s disease. **b** The patient was later diagnosed as intestinal tuberculosis

The number of ulcers ranged from 1 to 10. Six patients in the resolved group and 2 patients with progression had more than 1 ulcer (30% vs. 50%; *p* = 0.485). The ulcer sizes ranged from less than 0.5–2 cm. Three out of 4 patients with progression had ulcers larger than 1 cm, while only 3 out of 20 resolved patients had ulcers larger than 1 cm (75% vs. 15%; *p* = 0.035). Ulcer with irregular fold was found in 4 patients (Fig. 2), one in the resolved group and three in those with progression to significant disease (5% vs. 75%; *p* = 0.008). Insertion of terminal ileum was performed in 17 out of 24 patients (70.8%) and terminal ileum involvement was observed in 6 patients, two in the resolved group and 4 in those with progression (15.4% vs. 100%; *p* = 0.006). The median time from discovery of asymptomatic cecal ulcers to spontaneous endoscopic remission was 24 months (ranging from 12 to 84 months) in the resolved ones. The median endoscopic follow-up time was 36 months in resolved patients and 47.5 months in those with progression (Table 2).

**Table 2:** Endoscopic characteristics of patients with asymptomatic cecal ulcers

	Resolved (n = 20)	Progression to disease (n = 4)	*p* value
Endoscopic finding			
Ulcer number > 1	6 (30%)	2 (50%)	0.578
Ulcer size (cm)	0.40 (0.2–1.2)	1.75 (0.2–2)	0.088
Ulcer size ≥ 1 cm	3 (15%)	3 (75%)	0.035**
Ulcer with irregular fold	1 (5.00%)	3 (75.00%)	0.008**
Terminal ileum involvement	2 (15.38%)	4 (100.00%)	0.006**
Time to spontaneous endoscopic remission (months)	24.00 (12–84)	NA	
Longest endoscopic follow up period (months)	36.00 (14–119)	47.50 (12–65)	0.961

Continuous data are expressed as median (Range). Categorical data are expressed as number and percentage

*NA* not available

**p < 0.05

### Clinical course and outcome

Among the 20 patients with spontaneous remission, the histology results were mainly non-specific. Among them, 6 patients underwent a second follow-up colonoscopy, and one patient had a third follow-up endoscopy. In all 7 of these patients, the ulcer remained resolved in the subsequent endoscopy. The median time of the longest endoscopic follow-up in these patients was 63 months (range 36–119 months).

One patient was diagnosed with intestinal tuberculosis, based on the finding of granulomatous inflammation and positive acid-fast stain bacilli in histological study. In the second patient with intestinal tuberculosis, the histologic result was chronic active follicular colitis without granuloma, basal lymphoplasmacytosis or glandular distortion. Cecal and terminal ileum ulcers persisted during the second and third endoscopic exams, which were conducted 6 months and 18 months after the first diagnosis. The histologic results remained inconclusive. Cultures for Mycobacterium tuberculosis were also negative. Anti-tuberculosis was administered on evidence of a positive quantiFERON test and persistent ulceration, even though the patient remained asymptomatic. The lesions resolved completely after treatment with antituberculosis medications.

The third patient developed body weight loss and right lower quadrant abdominal pain 3 years after the first diagnosis of cecal and terminal ileum ulcers. The repeated colonoscopy showed persistent ulcerations confined over cecum and terminal ileum, without much progression. Crohn’s disease was diagnosed finally in the third endoscopy (4 years after the initial endoscopic exam with cecal ulcers) on the basis of crypt distortion and the presence of cryptitis, crypt abscesses and a complete survey including lab tests and abdominal computed tomography that excluded other possibilities.

The fourth patient presented with peri-appendiceal shallow ulcers as well as ulcers in the terminal ileum. She was well until 3 years later when bloody stool occurred. Ulcerative colitis was diagnosed based on proctitis with typical shallow ulcers and persistent peri-appendiceal and terminal ileum involvement.

## Discussion

Studies specifically on the long-term outcomes of cecal ulcers in asymptomatic patients are scarce. Our study demonstrated that clinically significant diseases, including colonic tuberculosis, ulcerative colitis and Crohn’s disease could develop in a portion of patients with asymptomatic cecal ulcers. The endoscopic features that predict a clinically significant disease include ulcers larger than 1 cm, terminal ileum involvement and ulcers with irregular fold.

Previous reports have focused on symptomatic (such as fever or lower gastrointestinal bleeding) patients with cecal ulcers [1, 8–10]. One case series from India reported 58 symptomatic patients who had cecal ulcers with or without ileum involvement [10]. The most common etiology was non-specific ulcers or infectious nature. There were no endoscopic features that clearly identified ulcers with a specific cause.

The differential diagnosis of cecal ulcer depends largely on the nature of the patient population. Without symptoms of acute illness, such as fever and abdominal pain, the causes may be narrowed down to chronic inflammatory diseases. In our cohort, none of the patients reported gastrointestinal symptoms. Moreover, all 24 patients had a normal white blood cell count and hemoglobin level. Only one patient with colonic tuberculosis had an elevated C-reactive protein level (4.47 mg/dL). Guaiac-based fecal occult blood test was trace positive in only one patient and negative in the rest. This observation was consistent with a previous report by Rodriguez-Lago et al. showing that 23% of patients had a negative fecal immunochemical test 2 years before inflammatory bowel disease was diagnosed [11]. Early detection of preclinical disease may be the reason that the cases in our study had normal laboratory data. In such cases, the endoscopic features of asymptomatic patients with a significant disease may not be as typical as those of their symptomatic counterparts. For example, one report of 69 patients with intestinal tuberculosis showed that up to 32.8% presented with ileocecal valve deformities and as high as 44.8% patients presented with luminal stenosis, either in the terminal ileum or other parts of the intestine [12]. In our two cases, the endoscope could pass through the ileocecal valve without resistance and other specific findings of intestinal tuberculosis, such as patulous ileocecal valve, were not observed [12, 13].

The diagnosis of intestinal tuberculosis is typically made by the evidence of a typical histological picture. For example, granuloma could be found in up to 73% of patients with a diagnosis of intestinal tuberculosis (50 out of 68 patients) [12]. In our cases, the first patient had typical histological findings in the initial endoscopic exam, including granulomatous inflammation and positive acid-fast stain bacilli. In the second case, however, the diagnosis was obscure. The endoscopic biopsies were performed 3 times and results were all inconclusive. Tuberculosis cultures of endoscopic samples were also negative. The diagnosis was made based on positive quantiFERON test and complete remission after antituberculosis medications.

Atypical presentation was also reported in the early course of ulcerative colitis. A previous study reported that up to 19.2% (46 out of 240 cases) of ulcerative colitis cases had an atypical distribution, such as appendiceal orifice inflammation in the initial endoscopic exam [14]. The case of ulcerative colitis in our study had only peri-appendiceal and terminal ileum ulcers initially. The diagnosis was not made until 3 years later when bloody stool occurred and the presence of a typical endoscopic picture of proctitis was observed.

Several endoscopic features have been reported to differentiate Crohn’s disease from intestinal tuberculosis, such as longitudinal ulcers, and cobblestone appearance [13, 15]. The case with Crohn’s disease in our study did not show these two typical endoscopic characteristics and the location of the involved area was confined to the cecal and terminal ileum. It may again represent a case in which an early natural course was discovered due to the initial screening colonoscopy.

### Limitations

There are a few limitations in this study. First, the case numbers were relatively low. However, the prevalence of both asymptomatic inflammatory bowel disease and intestinal tuberculosis in our cohort was in line with previous studies. The prevalence of asymptomatic inflammatory bowel disease was 0.056–0.355% in Western countries and 0.06% in Japan in individuals participating in colon cancer screening [4, 11, 16–18]. In our cohort, the prevalence of inflammatory bowel disease was 0.059% (2 cases out of 34,036 persons), which is very similar to the rate seen in Japan. Since we focused on cecal ulcers only, the number of patients with ulcerative colitis could be underestimated. Besides inflammatory bowel disease, intestinal tuberculosis was diagnosed in 2 cases out of 34,036 asymptomatic persons in our study. This prevalence was also close to the prevalence in Japan (5 cases in 236,000 persons) [4]. Second, 8 patients refused to have a follow-up colonoscopy in the out-patient department. The main reason for refusal was that the patients felt well and did not have any relevant symptoms. Without follow-up, we could not determine their subsequent outcome. Nonetheless, a previous report showed that 36% of patients with a diagnosis of asymptomatic inflammatory bowel disease developed symptoms in an average of 25 months [11]. The clinical follow-up duration of these 8 patients was 49.1 months (range 19–99 months) in the out-patient department. Without further development of symptoms, we speculate that the lack of follow-up data for these patients may have little clinical significance. Third, the prevalence of both intestinal tuberculosis and inflammatory bowel disease differs among regions, and therefore the results of this study might not be representative of other countries.

## Conclusion

In conclusion, patients with an asymptomatic cecal ulcer may develop diseases such as colonic tuberculosis, Crohn’s disease and ulcerative colitis. Larger ulcer size (> 1 cm), terminal ileum involvement and ulcers with irregular fold were three endoscopic risk factors associated with clinically significant diseases. When the above features are present, patients should be closely monitored even if they are asymptomatic.

## Data Availability

The datasets used and/or analyzed during the current study will be available from the corresponding author on reasonable request.

## Abbreviations

NSAID: Nonsteroidal anti-inflammatory drug
OR: Odds ratio
WBC: White blood cell
CRP: C-reactive protein
FOBT: Fecal occult blood test
NA: not available
